# Erratum: “Following the molecular motion of near-resonant excited CO on Pt(111): A simulated x-ray photoelectron diffraction study based on molecular dynamics calculations” [Struct. Dyn. **2**, 035102 (2015)]

**DOI:** 10.1063/1.4958888

**Published:** 2016-07-18

**Authors:** Michael Greif, Tibor Nagy, Maksym Soloviov, Luca Castiglioni, Matthias Hengsberger, Markus Meuwly, Jürg Osterwalder

**Affiliations:** 1 Departement of Physics, University of Zürich, Winterthurerstrasse 190, CH-8057 Zürich, Switzerland; 2 Department of Chemistry, University of Basel, Klingelbergstrasse 80, CH-4056 Basel, Switzerland; 3 IMEC, RCNS, Hungarian Academy of Sciences, Magyar tudósok körútja 2, HU-1117 Budapest, Hungary

The manuscript “Following the molecular motion of near resonant excited CO on Pt(111): A simulated x-ray photoelectron diffraction study based on molecular dynamics calculations” (Ref. 1) contained two mistakes. They concern two technical points: (1) The field amplitude of the THz pulse was 12 × 10^8^ V/m in all simulations and not 6 × 10^8^ V/m. (2) In the presented simulations, the pulse was not turned off after 5 ps.

As a consequence, all simulations (50 independent MD simulations) were repeated with a field strength of 12 × 10^8^ V/m and with turning off the excitation pulse correctly after 5 ps. Analysis of these trajectories demonstrates that relaxation of the frustrated “wagging-type” translation does take place after the excitation pulse is turned off (see new Figure 4). In addition, Figures 3, 5, and 6 were redrawn. The new simulations do not affect the conclusion that the frustrated translation is sufficiently excited to allow direct detection via the forward scattering peak in a photoelectron diffraction experiment. In addition, the analysis agrees much better with the intuitive expectation that, after the excitation pulse is turned off, the amplitude of the frustrated translation relaxes exponentially.

**FIG. 3.**
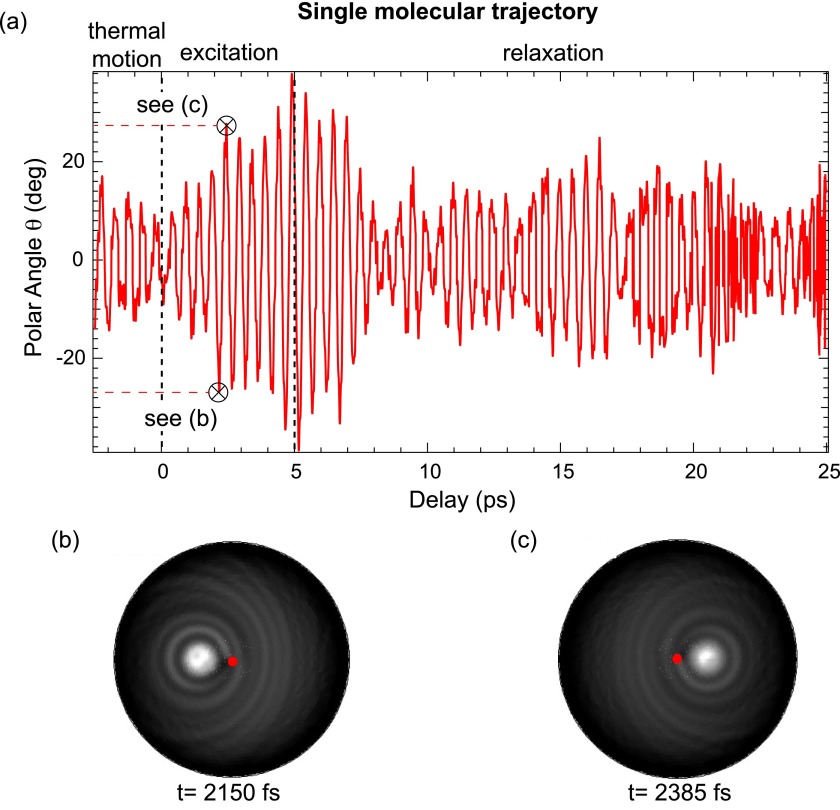
(a) Time evolution of the wagging motion of a CO molecule on a Pt(111) surface for a single molecule before, during and after excitation with a 2 THz radiation pump pulse. Polar angles of the carbon-to-oxygen direction are plotted, irrespective of the azimuthal direction. For delays between the dashed vertical lines, the pump pulse is present. Full XPD maps are displayed in (b) and (c), corresponding to times as marked in (a). The specific delays are chosen to show the near maximum excursions of the polar angle. The two maps are plotted in stereographic projection in a linear grey scale with maximum intensity in white. The red dot indicates emission normal to the surface.

**FIG. 4.**
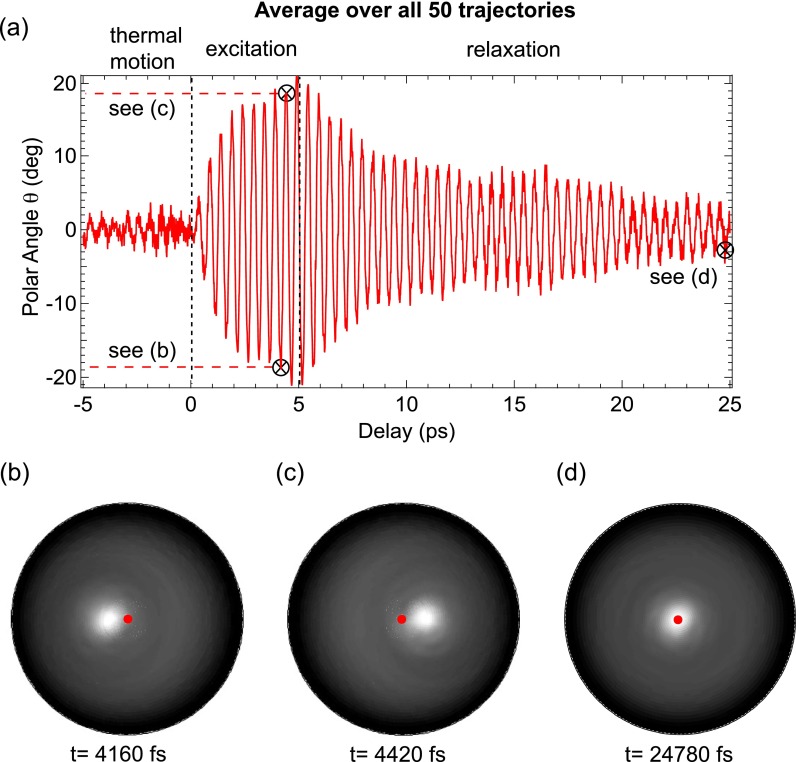
Simulation of a THz pump-XPD probe measurement. In (a), the time-dependent polar angle of maximum emission from the averaged diffraction maps is plotted. It represents the ensemble-averaged orientation of the CO molecules on Pt(111). At delays between the dashed lines, the pump pulse is present. During this time, an oscillation with *ν* = 2.0 THz grows continuously and relaxes again thereafter. (b)–(d) The averaged diffraction maps that were calculated with SSC simulations. No normalization was applied. The delay times of all three patterns are marked in (a) with black circles. Each diffraction map is ensemble averaged over calculations from 50 different cluster files. The red dots in the center of the maps show the normal emission direction.

**FIG. 5.**
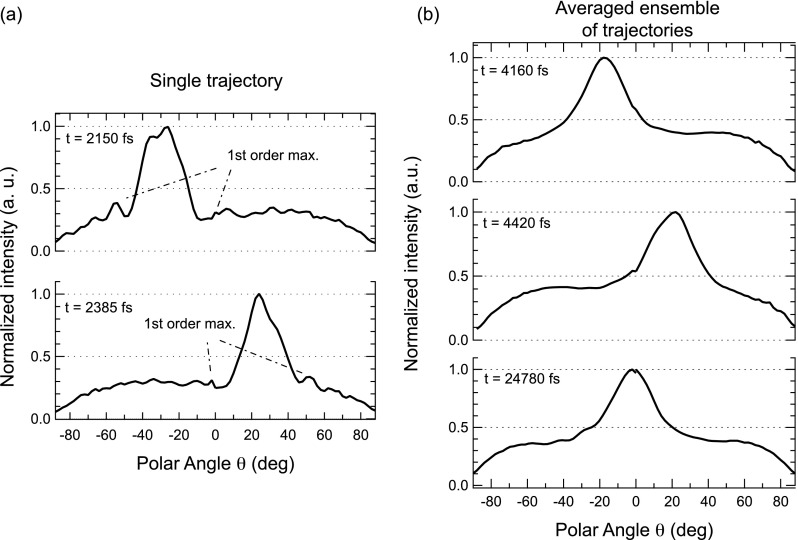
Polar intensity scans for quantifying the angular contrast. Panels (a) and (b) show sections along the x direction through the simulated photoelectron diffraction maps displayed in Figs. 3 and 4, respectively. All curves have been normalized so that maximum intensity equals unity.

**FIG. 6.**
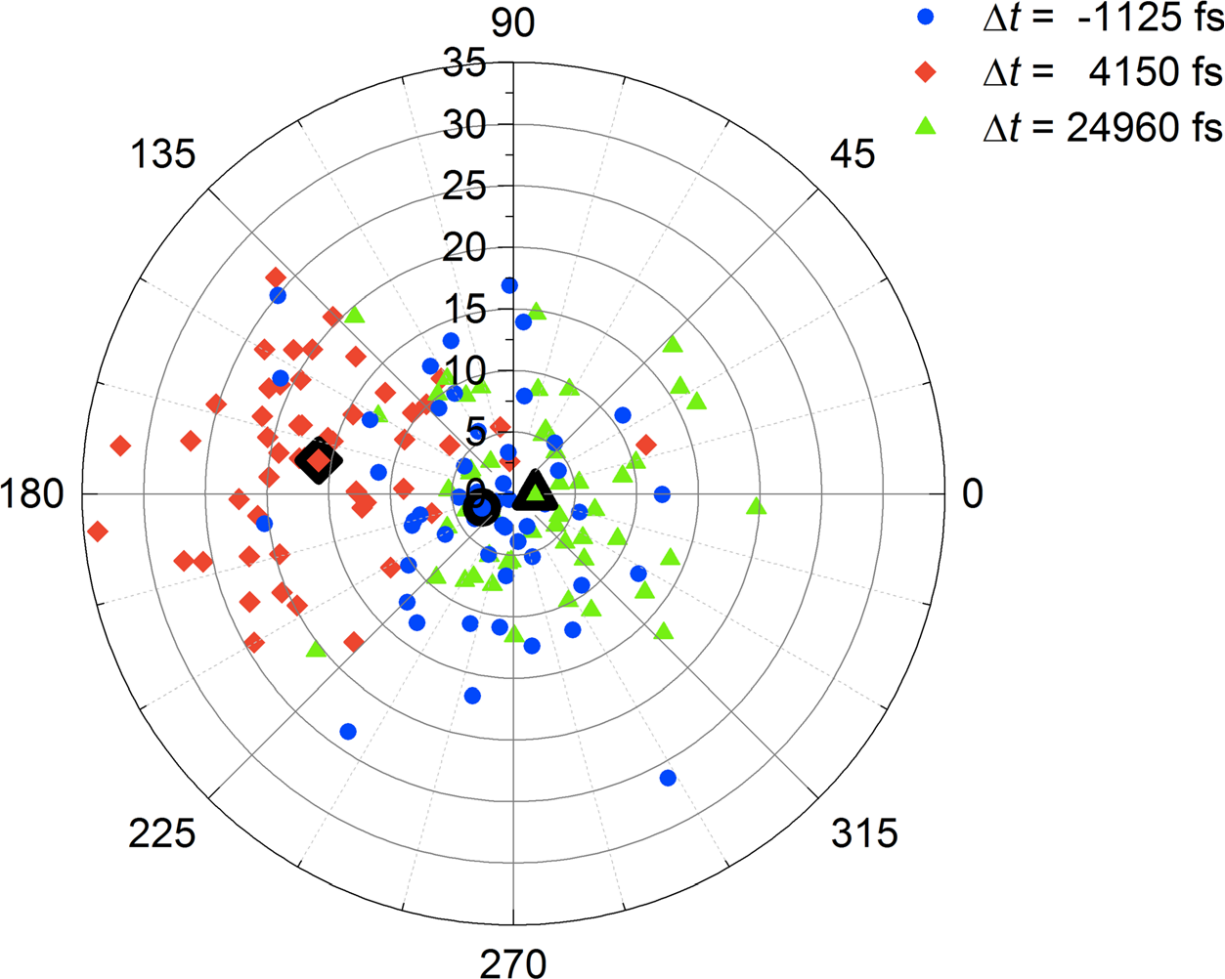
Angular order of the CO molecules. At three different times, the angular position (θ,ϕ) of the oxygen atom with respect to the carbon emitter is plotted for each of the 50 trajectories: before the THz pulse (black), near the end of the pulse (red), and 20 ps after the pulse (green). Data points highlighted with bold lines represent the centers of gravity of the respective distribution.

**Specific Changes:** All simulations were carried out with a field amplitude of 1.2 × 10^9^ V/m which induces motion parallel to the surface (hindered translation) by coupling to the point charges on the polar CO molecule.

In Fig. 3, the behavior of a single, individual CO molecule bonded at an atop site of a Pt(111) cluster is illustrated over a time interval that ranges from 2.5 ps before to 20 ps after excitation with the THz pulse. In (b) and (c), simulated C 1s XPD patterns covering the 2*π* solid angle above the surface are shown. In both plots, there is a broad and bright spot associated with the forward scattering peak along the carbon-oxygen direction.

After the pump pulse is turned off (*t* > 5 ps), the amplitude relaxes back to its characteristic level of thermal fluctuations within the first 10 ps. The present model does not include dissipation via electronic friction induced by the interaction between the vibrating CO-dipole and the electrons of the metal which would further damp the oscillations, but are not expected to be dominant over the direct vibrational coupling. We conclude that after excitation, the hindered translation remains activated for several vibrational periods which should be sufficient for detection in a real experiment.

The wagging amplitude in Fig. 3(a) shows hints of a beating pattern. This suggests that energy transfer between different modes of molecular motion via vibrational coupling might be observable. Such mode coupling can also be detected in the power spectra of the Pt-C, C-O, and Pt-C-O coordinates and the motion characterizing the frustrated translation. It is further corroborated by the observation that the frequency of the frustrated translation during and after excitation is 2.0 THz and 1.8 THz, respectively. In other words, after the harmonic perturbation is switched off, the frequency of the frustrated translation returns to its equilibrium value.

In a real experiment, XPD intensity modulations are averaged over an ensemble of molecules. To mimic this, the 50 diffraction patterns obtained from 50 independent trajectories were averaged at each time step and a molecular movie was created that shows the averaged time-evolution of the full ensemble. In Fig. 4, parts of the movie are presented. It shows a similar delay scan as in Fig. 3 covering 30 ps, where the 5 ps long pump pulse is “on” from 0 to 5 ps. Before and after this excitation period, the system is unperturbed.

In Fig. 4(a), the time series of average polar tilt angles of the CO molecules is shown. It was determined by extracting the emission maximum of the averaged diffraction maps obtained from the SSC calculations. Before the arrival of the THz pulse, the polar angle is centered around 0°, with much lower fluctuations than in the single trajectory of Fig. 3(a). Although individual molecules show an activated frustrated translational mode, the isotropic distribution of the azimuthal plane of oscillation as well as their incoherent phases lead to an averaging out of the polar tilt angle that is extracted from the averaged XPD maps.

The ensemble-averaged polar angle (see Fig. 4) shows that after switching off the THz field, the amplitude of the oscillations in the polar angle starts relaxing exponentially but shows hints of a beating. This can be explained by energy flow between the highly excited frustrated translational mode and the less excited frustrated rotational mode. These data suggest that one should be able to extract information about the temporal evolution of the energy transfer between different modes from such experiments.

Fig. 5 illustrates these observations more clearly, along with the disappearance of the interference fringes, by displaying polar sections along the x direction through the simulated diffraction maps. More importantly, the curves demonstrate that the diffraction contrast remains quite pronounced even after the ensemble averaging. The contrast can be quantified by the anisotropy of the forward scattering peak, defined as $A=(I_{max}-I_{ped})/I_{max}$, where *I_max_* is the maximum intensity of the peak and *I_ped_* is a mean value of the pedestal intensity to the left and right of the peak. While single trajectories (Fig. 5(a)) show values of typically about *A* = 0.7, ensemble averaging (Fig. 5(b)) reduces this to ≈0.63.

The temporal evolution of the azimuthal alignment is illustrated in Fig. 6, in which the polar and azimuthal positions of the oxygen atoms with respect to the carbon emitters are plotted for three different times before, during, and after the THz pulse. While the distribution of the 50 different molecular trajectories at delay Δ*t* = −1125 fs shows no preferred azimuthal alignment (bold face circle), the molecules have the highest azimuthal order at the end of the pump period, to which Δ*t* = 4150 fs is close (bold face square). This latter time is chosen such that the coherent wagging oscillation is close to its maximum amplitude. At times long after the pump pulse (bold face triangle), like, e.g., at Δ*t* = 24960 fs, the distribution has relaxed to its original form.

See supplementary material for the ensemble-averaged diffraction patterns that were recomputed (by O. T. Unke). The movie starts at −5 ps before the excitation pulse (which interacts with the sample between 0 and 5 ps) and lasts until 35 ps.

## References

[1] M. Greif , T. Nagy , M. Soloviov , L. Castiglioni , M. Hengsberger , M. Meuwly , and J. Osterwalder , Struct. Dyn. , 035102 (2015). 10.1063/1.4922611 26798798 PMC4711632

